# Editorial to the IJMS Special Issue “Aptamer-Mediated Cancer Theranostics”

**DOI:** 10.3390/ijms24087253

**Published:** 2023-04-14

**Authors:** Michael K. Danquah

**Affiliations:** Department of Chemical Engineering, University of Tennessee at Chattanooga, 615 McCallie Ave., Chattanooga, TN 37403, USA; michael-danquah@utc.edu

Aptamers have emerged as a new generation of bioaffinity probes with enhanced target binding specificity and selectivity. They possess high binding affinities, enabling various biomedical applications relating to molecular targeting and recognition [1,2]. Aptamers are non-viral oligonucleotides and can be generated via the Systematic Evolution of Ligands by Exponential Enrichment (SELEX). Recent aptamer research applications have focused on the use of aptamers in cancer diagnostics and targeted therapy due to their attractive biophysical and biochemical features including bioavailability, biocompatibility, stability, and low immunogenicity [3]. A major advantage of aptamer-based cancer theranostics is the ability to precisely target cancer cells and minimize off-target effects using highly specific aptamer ligands that can selectively bind to cancer cells and biomarkers [4]. As a result, aptamer-based theranostics offer a more effective and less toxic alternative. Aptamer-mediated approaches can be used to develop technologies for the early detection and diagnosis of cancer [5]. This is vital because early detection of cancer can significantly improve the chances of successful treatment. Aptamer-based biosensors and imaging agents can be used to detect cancer biomarkers in body fluids, such as blood and urine, providing a non-invasive and highly sensitive diagnostic tool [6,7]. Additionally, aptamer-based imaging agents can be used for the real-time monitoring of tumor growth and response to therapy. Aptamer-based targeted treatment has also shown promise to be able to overcome drug resistance in cancer cells. Resistance to chemotherapy is a major challenge in cancer treatment and it often leads to treatment failure and disease recurrence. Aptamer-based theranostics can overcome this challenge by delivering drugs directly to cancer cells and bypassing mechanisms of drug resistance [8]. Moreover, aptamers can be designed to target multiple biomarkers, making them effective against heterogeneous cancer cell populations.

The development of aptamer-based biomedical technologies for cancer therapy requires interdisciplinary approaches that involve chemistry, biology, and engineering. Researchers are continuously working to improve the stability, specificity, and delivery of aptamer-based theranostics. Novel aptamer conjugation techniques, such as click chemistry, have been developed to improve the stability and efficacy of aptamer–drug conjugates [9]. Additionally, new delivery systems, such as exosomes and cell-penetrating peptides, are being explored to improve the delivery of aptamer-based formulations. Despite the significant progress in aptamer-mediated cancer theranostics, there are still several challenges that need to be addressed. One of the major challenges is the lack of standardization and reproducibility of aptamer synthesis and characterization. This can hinder the translation of aptamer-based approaches from preclinical studies to clinical trials [10]. Moreover, the regulatory approval process for aptamer-based theranostics is still in its early stages, and further studies are needed to establish the safety and efficacy in humans.

The Special Issue ‘Aptamer-Mediated Cancer Theranostics’ presents a collection of research advances from researchers and practitioners on various applications of aptamers in emerging strategies for cancer treatment and diagnosis. These collections include: Development of HER2-Specific Aptamer–Drug Conjugate for Breast Cancer Therapy; Therapeutic Application of Drug-Conjugated HER2 Oligobody (HER2-DOligobody); Neuroblastoma GD2 Expression and Computational Analysis of Aptamer-Based Bioaffinity Targeting; The Potential of Aptamer-Mediated Liquid Biopsy for Early Detection of Cancer; and Advances in Oligonucleotide Aptamers for NSCLC Targeting.

In ‘Development of HER2-Specific Aptamer–Drug Conjugate for Breast Cancer Therapy’, the authors evaluated the effectiveness of a conjugate of HER2 RNA aptamers and the anticancer drug DM1 in HER2-overexpressing breast cancer models. The conjugate was prepared and analyzed, and its cell-binding affinity and cytotoxicity were determined using confocal microscopy and WST-1 assay. In vivo anti-tumoral efficacy was evaluated in mice carrying BT-474 breast tumors overexpressing HER2. The HER2-specific RNA aptamers showed specific and efficient binding to HER-positive BT-474 breast cancer cells, and the HER2-specific aptamer–drug conjugate showed strong toxicity to the target cells. The conjugate was also found to inhibit tumor growth more effectively in mouse xenografts of BT-474 tumors. This research suggests the HER2 aptamer–DM1 conjugate as a target-specific anti-cancer modality with enhanced effectiveness for HER2-overexpressing target tumors [11].

In ‘Therapeutic Application of Drug-Conjugated HER2 Oligobody (HER2-DOligobody)’, the authors developed DOligobody, a drug-conjugated oligobody comprising an aptamer–drug conjugate and an antibody for cancer therapy. Antibody–drug conjugates (ADCs) have reduced “off-target” side effects and enhanced therapeutic efficacy but have limitations such as difficulty in site-specific conjugation of payload and tissue penetration. Aptamers have advantages in drug delivery, as they can be easily and stably conjugated with cytotoxic drugs. The DOligobody comprising an anti-HER2 aptamer and monomethyl auristatin E inhibited the growth of HER2-positive NCI-N87 cells and reduced tumor growth in a xenograft mouse model. The results suggest that DOligobody may be a powerful platform for rapid, low-cost, and effective cancer therapy [12].

In ‘Neuroblastoma GD2 Expression and Computational Analysis of Aptamer-Based Bioaffinity Targeting’, the authors discussed the challenges of treating neuroblastoma (NB), a pediatric cancer with high mortality rates. Despite advanced treatment options, high-risk patients who achieve remission often experience relapse due to minimal residual disease (MRD). Disialoganglioside (GD2), a lipo-ganglioside found in NB tumor cells, represents a unique antigen for subclinical NB MRD detection and analysis to determine response to treatment. The article highlighted various analytical assays for GD2 detection and quantification, as well as computational approaches for GD2 characterization based on high-throughput image processing and genomic data analysis. Understanding GD2 expression and quantification in NB MRD can aid in developing more effective treatments for high-risk NB patients [13].

In ‘The Potential of Aptamer-Mediated Liquid Biopsy for Early Detection of Cancer’ the authors discussed the potential of aptamer-mediated liquid biopsy as a non-invasive tool for early cancer detection and monitoring of disease progression and treatment efficacy. Liquid biopsy has emerged as an exciting new technology for detecting cancer at an early stage. Aptamers, short oligonucleotides consisting of either DNA or RNA, are considered promising recognition ligands for liquid biopsy due to their unique properties. The paper covers various methods for isolating and purifying bio-analytes in liquid biopsy specimens and discusses the challenges and future potential for clinical applications [14].

In ‘Advances in Oligonucleotide Aptamers for NSCLC Targeting’, the authors discussed non-small-cell lung cancer (NSCLC), which is the most common type of lung cancer worldwide and has a high rate of resistance to currently available therapies. The authors propose the use of nucleic-acid aptamers, short single-stranded oligonucleotides that fold into high-affinity ligands toward disease-associated proteins, as a promising option for NSCLC early diagnosis and targeted therapy. They describe the SELEX (systematic evolution of ligands by exponential enrichment) process, which is used to generate aptamers and has the potential for novel biomarker discovery of both diagnostic and therapeutic interest. The authors review the most important applications of SELEX technology and aptamers for NSCLC handling, highlighting the potential to overcome obstacles faced by currently used therapeutic modalities [15].

## References

[1] Jeevanandam J., Tan K.X., Danquah M.K., Guo H., Turgeson A. (2020). Advancing aptamers as molecular probes for cancer theranostic applications—The role of molecular dynamics simulation. Biotechnol. J..

[2] Acquah C., Agyei D., Obeng E.M., Pan S., Tan K.X., Danquah M.K. (2020). Aptamers: An emerging class of bioaffinity ligands in bioactive peptide applications. Crit. Rev. Food Sci. Nutr..

[3] Hegde Y.M., Theivendren P., Srinivas G., Palanivel M., Shanmugam N., Kunjiappan S., Vellaichamy S., Gopal M., Dharmalingam S.R. (2023). A Recent Advancement in Nanotechnology Approaches for the Treatment of Cervical Cancer. Anti-Cancer Agents Med. Chem. (Former. Curr. Med. Chem.-Anti-Cancer Agents).

[4] He S., Du Y., Tao H., Duan H. (2023). Advances in aptamer-mediated targeted delivery system for cancer treatment. Int. J. Biol. Macromol..

[5] He F., Wen N., Xiao D., Yan J., Xiong H., Cai S., Liu Z., Liu Y. (2020). Aptamer-based targeted drug delivery systems: Current potential and challenges. Curr. Med. Chem..

[6] Vandghanooni S., Sanaat Z., Barar J., Adibkia K., Eskandani M., Omidi Y. (2021). Recent advances in aptamer-based nanosystems and microfluidics devices for the detection of ovarian cancer biomarkers. TrAC Trends Anal. Chem..

[7] Sa P., Sahoo S.K. (2023). Recent advances in aptamer-based nanomaterials in imaging and diagnostics of cancer. Aptamers Eng. Nanocarriers Cancer Ther..

[8] Li X., Wu X., Yang H., Li L., Ye Z., Rao Y. (2019). A nuclear targeted Dox-aptamer loaded liposome delivery platform for the circumvention of drug resistance in breast cancer. Biomed. Pharmacother..

[9] Ghasemi K., Darroudi M., Rahimmanesh I., Ghomi M., Hassanpour M., Sharifi E., Yousefiasl S., Ahmadi S., Zarrabi A., Borzacchiello A. (2022). Advances in aptamer-based drug delivery vehicles for cancer therapy. Biomater. Adv..

[10] McKeague M., Calzada V., Cerchia L., DeRosa M., Heemstra J.M., Janjic N., Johnson P.E., Kraus L., Limson J., Mayer G. (2022). The Minimum Aptamer Publication Standards (MAPS Guidelines) for De Novo Aptamer Selection. Aptamers.

[11] Jeong H.Y., Kim H., Lee M., Hong J., Lee J.H., Kim J., Choi M.J., Park Y.S., Kim S.-C. (2020). Development of HER2-Specific Aptamer–drug Conjugate for Breast Cancer Therapy. Int. J. Mol. Sci..

[12] Kim H.J., Sung H.J., Lee Y.M., Choi S.I., Kim Y.-H., Heo K., Kim I.-H. (2020). Therapeutic Application of Drug-Conjugated HER2 Oligobody (HER2-DOligobody). Int. J. Mol. Sci..

[13] Sabbih G.O., Danquah M.K. (2021). Neuroblastoma GD2 Expression and Computational Analysis of Aptamer-Based Bioaffinity Targeting. Int. J. Mol. Sci..

[14] Roy D., Pascher A., Juratli M.A., Sporn J.C. (2021). The Potential of Aptamer-Mediated Liquid Biopsy for Early Detection of Cancer. Int. J. Mol. Sci..

[15] Rotoli D., Santana-Viera L., Ibba M.L., Esposito C.L., Catuogno S. (2020). Advances in Oligonucleotide Aptamers for NSCLC Targeting. Int. J. Mol. Sci..

